# A Mini Review on pH-Sensitive Photoluminescence in Carbon Nanodots

**DOI:** 10.3389/fchem.2020.605028

**Published:** 2021-01-22

**Authors:** Cui Liu, Fang Zhang, Jiao Hu, Wenhui Gao, Mingzhen Zhang

**Affiliations:** ^1^Department of Biophysics, School of Basic Medical Sciences, Institute of Medical Engineering, Xi'an Jiaotong University Health Science Center, Xi'an, China; ^2^Hubei Key Laboratory of Environmental and Health Effects of Persistent Toxic Substances, Institute of Environment and Health, Jianghan University, Wuhan, China

**Keywords:** carbon nanodots, pH-sensitive, photoluminescence, pH sensing, mechanism

## Abstract

Carbon nanodots (C-dots) with sp^2^/sp^3^ framework and diameter of <10 nm contain abundant functional groups or polymers on their surface. C-dots have attracted immense attention because of their unique optical properties, excellent biocompatibility, facile preparation, and low cost. With these merits, C-dots have been used in a wide range of applications including sensing, bioimaging, catalysis, and light-emitting devices. C-dots exhibit good optical properties, such as tunable emission wavelength, good photostability, nonblinking, up-conversion emission, etc. Of note, C-dots show intrinsic pH-sensitive photoluminescence (PL), indicating their great potential for pH sensing, especially in biotic pH sensing. In this review, we systematically summarize the pH-sensitive PL properties and the pH-sensitive PL mechanism, as well as recent research progress of C-dots in pH sensing. The current challenges of pH-sensitive C-dots and their future research focus are also proposed here. We anticipate this review might be of great significance for understanding the characteristics of pH-sensitive C-dots and the development of photoluminescent nanomaterials with pH-sensitive properties.

## Introduction

As a novel carbon nanomaterial, carbon nanodots (C-dots) have attracted extensive attentions because of their small particle size (generally <10 nm), wide source of raw materials, low cost, and excellent physical and chemical properties (Esteves da Silva and Gonçalves, 2011; Ding et al., 2014; Lim et al., 2015; Yuan et al., 2016; Liu et al., 2019). Unlike the previous carbon nanomaterials, C-dots are mainly composed of sp^2^/sp^3^ hybrid carbon with abundant functional groups or organic polymers on their surface. The C-dots mentioned here refer to carbon nanoparticles with carbon as the main constituent element, including carbon dots, carbon nanoparticles, graphene quantum dots, carbon quantum dots, and carbonized polymer dots in previous reports. The unique photoluminescence (PL) properties of C-dots including tunable emission, nonblinking, and excellent photostability enable them great potential in wide applications ranging from photoelectric device, fluorescence sensing, to bioimaging and nanomedicines (Hong et al., 2015; Lim et al., 2015; Yuan et al., 2016). Moreover, one of their most intriguing property is their pH-dependent PL. As the pH changes, the PL spectra or intensity of C-dots would change accordingly (Dong et al., 2010; Pan et al., 2010a,b; Qiao et al., 2010; Zhu et al., 2011, 2013). It is well-known that pH plays key role in industrial, agricultural, environmental, and biomedical fields (Wencel et al., 2014; Yin et al., 2015; Lei et al., 2017). It is of great significance for both scientific research and real-word applications to monitoring pH in disease diagnosis, environmental examination, food and beverage analysis, and so on (Wu et al., 2014; Mani et al., 2017). pH-sensitive nanoprobes based on organic dyes and nanocarriers have been developed to overcome the limitations of photobleaching, phototoxicity, and interference from background autofluorescence of traditional organic fluorescent dye. However, it is still difficult to achieve long-term and biological damage-free pH monitoring due to the poor biocompatibility and large particle size of organic dyes and nanocarriers. C-dots with small size, excellent photostability, and good biocompatibility have the potential to provide solutions for pH sensing in environmental, pharmaceutical, and especially *in vivo* medical applications.

**Figure 1:**
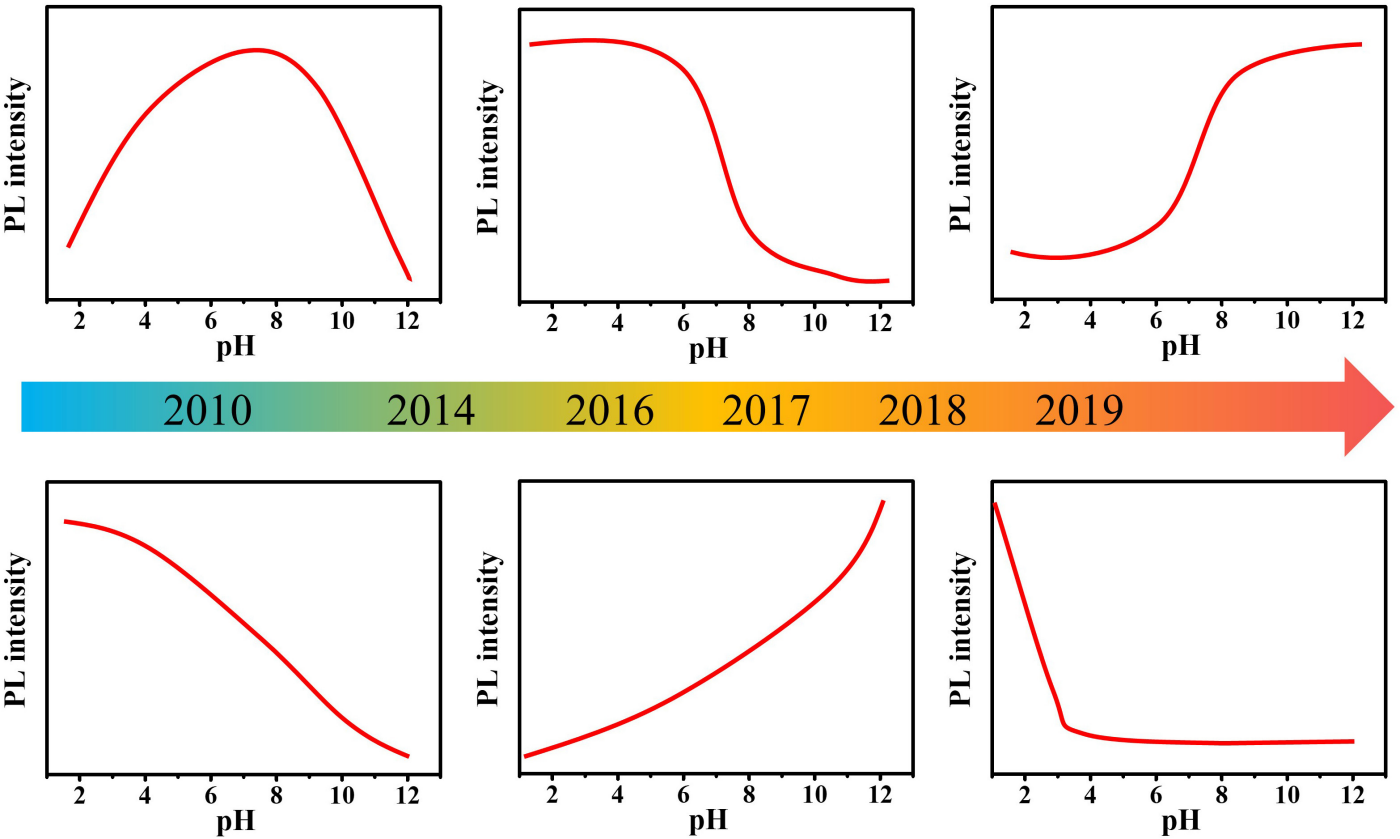
Timeline showing the pH-sensitive C-dots in the review.

In recent years, many synthetic methods have been developed to prepare pH-sensitive C-dots. Interestingly, although the synthesis methods, raw materials, structure, and morphology of C-dots are quite different, most C-dots possess pH-sensitive PL properties, despite their pH response behaviors are different (Figure 1) (Liu et al., 2007; Dong et al., 2010; Pan et al., 2010b; Kong et al., 2014; Lu et al., 2016; Hou et al., 2017; Sun et al., 2018; Pyne et al., 2019; Zhang M. et al., 2019). Mao et al. (Liu et al., 2007) prepared multicolor fluorescent C-dots from candle soot. When the pH increased from 3 to 13, the PL intensity first increased and then decreased with the optimal intensity appearing under neutral conditions. Chi's group (Dong et al., 2010) also prepared C-dots with similar pH sensitivity by acidic oxidation of activated carbon. Pan et al. (2010b) reported blue-emitting C-dots with PL intensity increased as the pH increased. Yang's group (Lu et al., 2016) obtained white fluorescent C-dots by a hydrothermal method. The PL intensity of C-dots gradually decreased as the pH increased from 0 to 14. Zhang M. et al. (2019) obtained N- and S-codoped C-dots with long emission wavelength by solvothermal method using l-cysteine and *o*-phenylenediamine as raw materials. The PL intensity of C-dots was very strong when pH value was between 1.0 and 2.0, but it suddenly decreased at pH ~3.0 and exhibited almost no PL in pH ranging from 8.0 to 13.0. It seems that the PL pH sensitivity of C-dots largely depends on the synthesis method, reaction conditions, raw materials, etc. The PL mechanism of C-dots pH sensitivity is still unresolved, resulting in the difficulty to effectively control the pH sensitivity of C-dots, which greatly limits their practical application. Although there are many reviews focusing on the preparation, properties, and applications of C-dots, reviews discussing the pH sensitivity of C-dot are still lacking. In this review, we mainly focus on the PL mechanism of the pH sensitivity of C-dots, including consistent and controversial conclusions. We hope that this review might provide a systematic understanding into the pH-sensitive PL of C-dots, thereby promoting the development of the application of pH-sensitive C-dots.

## pH-Dependent PL Mechanisms

**Figure 2:**
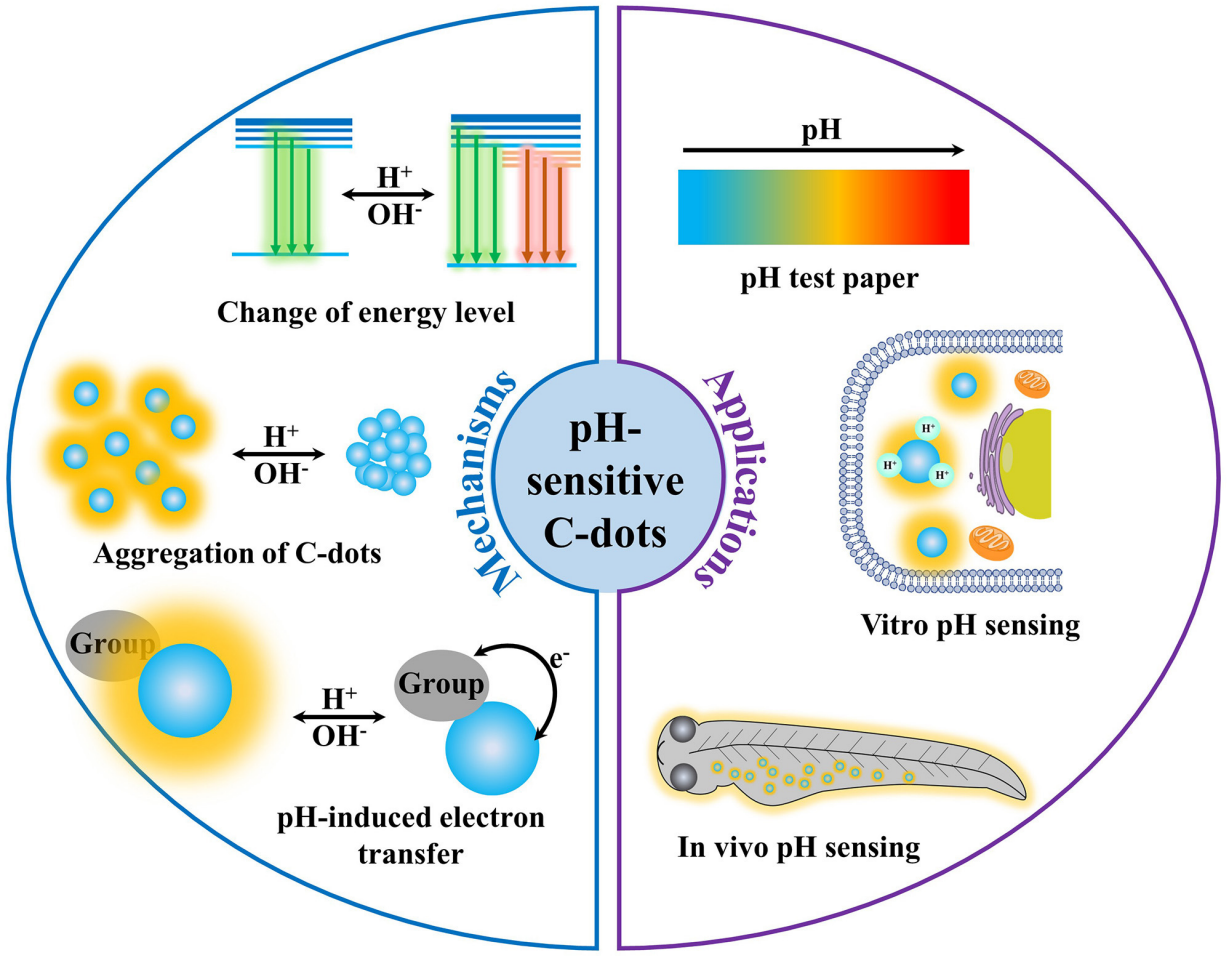
The mechanisms and applications of pH-sensitive photoluminescent C-dots.

The typical understanding of the mechanism of pH-sensitive PL mainly focused on the deprotonation and protonation of acidic and basic groups in ground or excited states, which could change the property and the rate of transition processes, and finally affect the PL spectrum and intensity of the fluorophores (Valeur, 2001; Lakowicz, 2006). For example, the lowest excited singlet state (*n*, π^*^) of salicylaldehyde would undergo an intersystem crossing process quickly (10^−7^–10^−9^ s), leading to emission of phosphorescence rather than fluorescence. However, when the phenolic hydroxyl group is deprotonated in an alkaline solution or the carbonyl group is protonated in a concentrated acid solution, salicylaldehyde exhibits strong fluorescence rather than phosphorescence. This is because the lowest excited singlet state of salicylaldehyde is (π, π^*^) rather than the (*n*, π^*^) when in the cationic or anionic forms. For C-dots, the structure is relatively complicated, mostly with graphite carbon or amorphous carbon as the main skeleton, and the surface is rich in carboxyl, hydroxyl, carbonyl, and other functional groups that may undergo protonation and deprotonation. This makes the pH-dependent PL mechanism of C-dots confusing and controversial. For example, Li et al. (2019) synthesized highly fluorescent C-dots by using tetrahydrofuran as carbon source via a hydrothermal treatment. As pH increased from 4 to 12, the PL intensity decreased linearly. They believed that the pH sensitivity of C-dots could be attributed to the reversible protonation and deprotonation of C-dots surface functional groups. However, there is no unified conclusion about the specific structure that should take responsibility to the pH sensitivity of C-dots. Currently, several mechanisms were proposed to interpret the pH-sensitive PL of C-dots (Figure 2), but hitherto there is still nothing that can match all the characteristics.

## Change of Energy Level

The pH sensitivity of C-dots originating from the protonation and deprotonation of oxygen-containing groups on the surface, which could change the energy level of C-dots, is one of the most comprehensively accepted mechanism. Wang et al. (2018) prepared C-dots with colorimetric and fluorescent pH-sensitive property. The PL peak of C-dots was at 630 nm in pH ranging from 1 to 6 and blue shifted to 590 nm with intensity slightly decreased under pH ranging from 7 to 14. They claimed that as pH increased, the –COOH and –OH changed to –COO^−^ and –O^−^, resulting in the change of PL of C-dots. However, they did not explain how the deprotonation of COOH and –OH affected the PL of C-dots. As proposed by Pan et al. (2010a) the PL of C-dots originated from zigzag sites. When the pH value of the environment decreased, the carbonyl oxygen of the zigzag sites of C-dots underwent a protonation process, and the structure of the triplet carbene was destroyed, resulting in the PL intensity decrease. Jin's group (Sui et al., 2016) investigated ultrafast carrier relaxation dynamics in C-dots under different pH by femtosecond transient absorption spectra. They claimed that the distribution of π electron cloud density between the carbon backbone and surface states would be changed by the deprotonation of carboxyl at higher pH. Therefore, the excited electrons in alkaline environment could be more easily trapped by surface within 1 ps, leading to the increase of PL intensity. Shi et al. (2016) prepared blue fluorescent C-dots with the PL intensity significantly increased as pH increased from 3 to 11. They believed that the increase of PL intensity of C-dots was induced by the deprotonation of carboxyl groups, which promoted the formation of delocalized π bonds and increase of *n* electrons. Meanwhile, Dutta Choudhury et al. (2017) observed that the PL intensity of C-dots gradually increased as the pH increased from 1 to 6 and then decreased at higher pH along with a significant PL red shift. The PL decay curves of C-dots at pH 2.7 and 9.5 were recorded under excitation of 374 and 400 nm. The lifetime of C-dots under higher pH was longer than that under lower pH value, which was more pronounced under the longer wavelength excitation, confirming that these are different between the optical characteristics of emissive species in basic and acidic conditions. Combining structural characterization results, they claimed that the carboxyl group was decoupled from the emission sites and therefore did not affect the energy level of C-dots, but it would cause intramolecular or the intermolecular hydrogen bond and lead to strong vibrational coupling of the OH functional groups in the surface state of C-dots, which possibly leads to energy level broadening and accordingly to the occurrence of broad bands in the absorption spectra of C-dots at low pH. When the pH increases from 2.5 to 7.9, the carboxyl group gradually underwent a proton dissociation process, the effect of hydrogen bonds was gradually eliminated, and the PL intensity increases without PL spectrum change, whereas the phenolic hydroxyl group was coupled with emission sites, and its deprotonation would create a new surface state in C-dots with a lower band gap. When the pH value gradually increased from 7.9, the carboxyl group has been in deprotonation state, whereas the phenolic hydroxyl group gradually underwent a deprotonation process, resulting in a decrease in PL intensity with the spectrum red-shifting. However, Basu and Mandal (2019) have the opposite opinion. They performed time-resolved PL emission to study the photophysics of C-dots in different pH solutions. As they proposed, using λ_ex_ ≤ 350 nm, the core and high-energy edge states were excited, producing hole-electron excitonic charge carriers without any PL emission. The surface fluorophores and low-lying edge states could be excited at excitation wavelength λ_ex_ ~375 nm, inducing broad PL, which was dependent on the pH and excitation wavelength. At pH ≥ 5, which marks the deprotonation of –COOH, the edge state was quenched by the carboxylate anion. Although it is believed that the protonation and deprotonation of oxygen-containing groups would have a great impact on the energy level of C-dots, which significantly change their fluorescence properties, these are often controversial on the contributions of specific functional groups on pH-sensitive properties of C-dots.

In addition, the energy level change caused by H^+^/OH^−^ is also considered to be one of the mechanisms of pH-sensitive PL in C-dots. Kong et al. (2014) reported pH-sensitive C-dots with different surface modifications. In their hypothesis, the absorbed OH^−^ could passivate the surface defects of C-dots and create a surface modified protective shell layer, which make C-dots isolated and lowered down the nonradiative recombination rate, leading to the increase of PL intensity and blue-shifting of emission peak, whereas H^+^ could destroy the protective shell passivated by OH^−^, create new surface defects on C-dots, and enlarge the size of C-dots by prolonging the length of conjugated chain, resulting in PL quenching and red-shifting of emission peak. Moreover, Guo et al. (2018) and Liu et al. (2017) also attributed the pH-dependent PL to the protective shell formed by the passivation of absorbed OH^−^ on the surface detects of C-dots. Nevertheless, Jia et al. (2012) believed that the protonation and deprotonation of the carboxyl groups on the surface of C-dots might induce electrostatic doping/charging to the C-dots and shift the Fermi level. Tan et al. (2018) also believed that the deprotonation of oxygen-containing functional groups on the surface of C-dots at high pH leaded to the increase of surface negative charge, which caused the shift of Fermi level and restrained the electron transition of some emission centers by occupying the energy levels. Zhao et al. (2019) believed that the H-bond could lead to the strong vibration coupling of –OH at lower pH, which caused the energy level broadening and increase the conformational rigidity of C-dots, bringing about the strong PL intensity. However, alkaline conditions could eliminate H-bonding effect through the deprotonation progress, resulting in the decrease of vibrational coupling and more discrete energy levels, giving rise to the decrease of PL intensity of C-dots. The C-dots reported by Hu et al. (2017) exhibited two emission centers at 550 and 630 nm attributing to N-state and P-state, respectively. As pH varied from 2 to 12, the intensity of peak at 550 nm was decreased significantly, whereas the intensity of peak at 630 nm exhibited continuous enhancing behavior, producing a linear relationship between the PL intensity ratios of the two emission peaks (*I*_550_/*I*_630_) and pH. As they claimed, the protonation of amine groups would dismiss the unpaired electrons and reduce the electron density in CDs, resulting in the decrease of PL intensity with blue shift of PL peak of N-state. The pH variation had little effects on the energy level of the P-state; the peak at 630 nm remained stable over a wide pH range of 2 to 12. The combination of P-related groups with OH^−^ radicals at high pH would enhance the density of the P-state, promoting the emission at 630 nm.

## Aggregation of C-Dots

There are also many researchers who believed that the pH sensitivity of C-dots was caused by the pH induced aggregation of C-dots. Xu et al. (2015) studied the reversible photophysical properties of C-dots at different pH. The UV-vis absorption and PL spectra of the C-dots exhibited reversible changes as the pH varied in the range of 3 to 13. As pH increased from 3 to 13, the absorption peak centered at 280 nm was red-shifted to 285 nm, whereas the absorption band at 220 nm gradually became ill-defined and finally disappeared. As the pH switched from 3 to 7, the emission peak of C-dots was red-shifted ~20 nm with increase in PL intensity. When the pH increased from 7 to 13, the PL intensity decreased, but the emission peak was stable. As the pH was restored to 3, both of the absorption and emission bands recovered their original shapes. Moreover, both of the optimal emission and excitation peaks of C-dots were red-shifted as the pH increased from 2 to 13. They considered that under acidic conditions, the C-dots gathered into lager particles rapidly, and oxygen-containing functional groups on the surface were slowly oxidized, leading to the PL decrease with blue-shifting. While under alkaline conditions, the structural tautomer occurred very fast, and the hydrogenation/deoxygenation process happened slowly, resulting in PL intensity decrease. Wang et al. (2015) proposed that as the pH increased, the aggregation C-dots appeared because of noncovalent molecular interactions, such as hydrogen bonds between the carboxyl groups, resulting in PL quenching. Mondal et al. (2019) reported blue emissive C-dots with PL intensity increase as pH increased from 1 to 7. They attributed the PL quenching in lower pH to the aggregation of C-dots induced by the protonation of carboxyl groups. Sun et al. (2018) explained pH sensitivity of C-dots by zeta potential and transmission electron microscopy (TEM) measurement. The zeta potentials of C-dots were ~17 mV at pH 4–5. As pH increased from 5 to 11, the zeta potentials decreased from 17 to −12 mV, along with the aggregation of C-dots shown in TEM images. So, they believed that under lower pH conditions, the protonation of carboxyl of surface of C-dots and noncovalent molecular interaction, such as hydrogen bonds, induced the aggregation of C-dots, resulting in PL quenching.

In addition to carboxyl groups, amides, amino groups, and phosphorus-containing functional groups may also undergo protonation and deprotonation, causing aggregation of the C-dots. Liu's group (Ye et al., 2019) attributed the PL pH response of C-dots to the aggregation–disaggregation induced by the deprotonation and protonation of the amino group on the surface of C-dots. Under alkaline conditions, because of the formation of intermolecular hydrogen bond, the C-dots might aggregate and result in PL quenching. On the other hand, Ren's group (Chen et al., 2019) believed that it might be due to the protonation of the amine groups on the surface of C-dots under strong acid conditions, leading to the aggregation of the C-dots and quenching their PL. At the same time, the new surface state (–${\text{NH}}_{3}^{+}$) would cause a slight blue shift in the emission spectrum as the pH rises. In fact, whether the protonation or the deprotonation of amino leads to the aggregation of the C-dots depends on their structure and electric charge. The C-dots often contain other negatively charged functional groups besides the amino. The C-dots with high content of amino have a positive charged surface and are stable under acidic conditions. When under alkaline conditions, the deprotonation of amino groups would result in the decrease of the amount of charge of C-dots and cause the aggregation. In contrast, the C-dots with less amino have a negatively charged surface. Under acidic conditions, the amino group is protonated, reducing the amount of charge of C-dots, and the aggregation of C-dots occurs. Su et al. (2019) synthesized nitrogen- and phosphorus-codoped C-dots. The C-dots exhibited blue fluorescent emission and green room temperature phosphorescence (RTP). Both of the fluorescence and RTP of C-dots exhibited pH sensitivity. The fluorescence decreased as pH increased from 9.15 to 13.55, whereas the RTP was more sensitive to pH in a wider linear ranging from 2.29 to 13.55. As proposed by the authors, the C-dots and phosphoric acid were connected by C–O–P, C–P, and N–P bonds. The protonation and deprotonation of the P–O bond mainly determined the fluorescence and phosphorescence pH sensitivity. Under acidic conditions, there were P–O–H groups on the surface of C-dots, and thus intramolecular and intermolecular hydrogen bonds are formed between P=O and –OH, which ensures the stability of C-dots. After adding NaOH, the deprotonation of the P–O–H bond happened, resulting in the dissociated of hydrogen bonds. Therefore, the steady surface state changed, and formation of crystal nucleus occurred. As the concentration of OH^−^ increased, the crystal nuclei begin to grow and cause the C-dots to gradually precipitated, resulting in the concentration of C-dots decrease in the solution. Consequently, both the RTP and fluorescence of C-dots ultimately decreased.

## pH-Induced Electron Transfer

In addition to changing the energy level, protonation, and deprotonation of functional groups could also cause electron transfer between functional group and emission site of C-dots as pH changes, thereby resulting in the PL change. Lv et al. (2019) reported the design and fabrication of pH-sensitive C-dots with strong orange light emission. They attributed the pH sensitivity of C-dots to the protonation of amino on the surface of C-dots, which enhanced the intramolecular electron transfer ability between –${\text{NH}}_{3}^{+}$ and C-dots, giving rise to the PL quenching. As the pH value reduced from 7.0 to 3.0, the amino groups gradually underwent a protonation process, resulting in the decrease of PL intensity of C-dots with a linear relationship in pH ranging from 6.5 to 3.0, whereas in the pH value range of 7.0–9.0, the PL intensity of C-dots changed negligibly because of the low protonation of the amino group. Liu H. et al. (2019) prepared fluorescent ratiometric pH-sensitive C-dots. As pH increased from 2.99 to 10.02, the absorption of C-dots was blue-shifted from 510 to 433 nm. The absorption ratio (*I*_433_/*I*_510_) of C-dots exhibited good linearity with the pH in the range of 5.4 to 8.0, and the pKa value was calculated to be 6.89. At the same time, the PL intensity of C-dots increased gradually with PL spectrum blue-shifting from 625 to 565 nm. They attributed the shift of PL spectra to the protonation and deprotonation of the pyridinic group, which could alter the internal charge transfer (ICT) process of C-dots. Meanwhile, green-emissive C-dots with pH sensitivity were synthesized by Wang Q. et al. (2019) via using m-phenylenediamine as the carbon source in the presence of strong H_2_SO_4_. The PL intensity of C-dots decreased obviously without any change in PL emission spectra as pH increased from 3.0 to 12.0. There were no obvious changes in UV-vis spectra and average sizes (according to the TEM images) of C-dots with pH values increasing from 6.0 to 10.0. Therefore, they excluded the aggregation-induced PL quenching and attributed the pH-sensitive PL of C-dots to the photoinduced electron transfer process from the electron lone pair in amino group to the C-dots. At high pH, the excited electrons of C-dots can transition from the highest occupied molecular orbital (HOMO) to the lowest unoccupied molecular orbital. Because of the fact that the HOMO energy level of amino group is higher than that of C-dots, the ground state electrons of the amino group could jump to the HOMO of C-dots, resulting in significant PL quenching.

## Applications of pH-Sensitive C-Dots

In the past decade, C-dots with superior optical properties, low toxicity, good dipersibility, and low cost have been used in detection, bioimaging, catalysis, optoelectronics devices, etc. (Baker and Baker, 2010; Esteves da Silva and Gonçalves, 2011; Li et al., 2012; Shen et al., 2012; Ding et al., 2014; Hola et al., 2014; Wang et al., 2014; Lim et al., 2015) Particularly, C-dots with the pH sensitivity have significant potential in pH sensing (Figure 2). Chen's group (Wang et al., 2019) prepared green fluorescent C-dots by solvothermal method using m-diaminobenzoic acid as the carbon sources. The PL intensity of C-dots increased as pH increased from 4.5 to 12. Because the PL intensity was linearly correlated with pH ranging from 6.0 to 9.0, the C-dots were applied to measure the pH of eight kinds of mineral water samples with pH ranging from 5.9 to 8.1. The pH values measured by pH-sensitive C-dots were consistent with that measured by a pH meter, proving the feasibility of pH sensors based on C-dots. Lü's group (Zhang T. Y. et al., 2019) synthesized tricolor emission C-dots *via* hydrothermal method using 5-amino-1,10-phenanthroline and citric acid. At pH 1, the PL of C-dots was relatively weak with a broad peak at 510 nm. When the pH increased to 2, the C-dots showed three strong emission peaks at 440, 510, and 620 nm. As the pH increased, the PL intensity of the three emission peaks gradually decreased with different extents. The C-dots were used as pH test paper to detect the pH value ranging from 1 to 14. The C-dots–based pH test paper showed bright green at pH 1 and gradually turned gray as the pH value increased from 1 to 8. Then, the color of the test paper changed from blue to purple when the pH increased from 8 to 13 and changed to yellow when the pH was 14. The color change from bright green to blue purple was very obvious. The pH value of the solution can be clearly distinguished with the naked eye thorough the color of the pH test paper. Besides, A novel orange-emissive C-dots were synthesized by Zhou's group (Yang et al., 2019) via microwave-assisted heating of 1,2,4-triaminobenzene and urea aqueous solution. The C-dots showed unique colorimetric and fluorescence response to pH changes. Because the PL intensity of C-dots exhibited a sigmoidal logistic relationship with pH over a wound-relevant pH range (Pan et al., 2010a,b; Qiao et al., 2010; Hong et al., 2015; Liu et al., 2019), medical cotton cloth was chosen to immobilize C-dots through hydrogen bond interaction, producing a novel pH indicator (i.e., C-dots–coated medical cotton cloth) with PL emission at 560 nm. The novel pH indicator not only has excellent biocompatibility and drug compatibility, but also has excellent leachability and high reversibility, contributing to its safety and reliability for wound pH sensing. Importantly, the use of C-dots–coated cloth to detect pH would not be disturbed by blood contamination and long-term storage, providing wound pH monitoring and long-term observations through visual response and quantitative determination in the case of blood contamination.

Meanwhile, because of their good biocompatibility, small size, and photostability, the pH-sensitive C-dots also were applied for pH sensing in organelle, cell, tissue, and living animals (Liu et al., 2018; Xia et al., 2018; Zhang et al., 2018; Ye et al., 2019; Zhang T. Y. et al., 2019). Huang's group (Zhang et al., 2018) prepared emerald emissive C-dots using the functional preservation strategy by simply mixing p-benzoquinone and ethanediamine at room temperature. The C-dots with abundant protective amino groups exhibited good hydrophilicity, which ensures good lysosomal targeting specificity and sensitive and selective detection of lysosomal pH changes *in vitro*. The PL intensity of CDs decreased as pH decreased from 7.40 to 3.00 with good linearity in the pH range of 4.00–5.80, which was beneficial for lysosome pH sensing. The C-dots were used to monitor pH changes in lysosomal during apoptosis of living cells. After incubation for 80 min, a strong green PL of C-dots was observed in normal A549 cells. However, when A549 cells were treated with dexamethasone, which can induce apoptosis in living cells due to proton leakage, their PL intensity showed time-dependent decrease, suggesting that the bright green C-dots can monitor the pH changes of lysosomal in real time. Liu's group (Ye et al., 2019) fabricated two photofluorescent C-dots, which showed a pH-sensitive red fluorescence in pH ranging from 1.0 to 9.0 with a linear range of 3.5 to 6.5, which is desirable for tracking the pH value in living cells. The MCF-7 cells were selected to verify the capability of C-dots to monitor the intracellular pH fluctuation. When the intracellular pH increases from 4.0 to 8.0, the red fluorescence of C-dots in cells decreased obviously. Furthermore, they examined the pH imaging of renal tissue slice and tumors with the C-dots. The renal tissue slice of the mouse treated with acetazolamide, which can neutralize the acid in tissue, exhibited apparently weaker fluorescence than that of the normal mouse. Because of tumor tissues having an acidic extracellular pH (pH 6.5–6.8), the tumor tissue slice showed a stronger fluorescence than normal muscle tissue (pH 7.4). Moreover, the pH-sensitive C-dots were administered to zebrafish in buffer solution with different pH ranging from 4.0 to 8.0. The zebrafish exhibited extremely weak emission under alkaline conditions (pH 8.0) and a strong red fluorescence signal under acidic conditions (pH 4.0). All these reports show that C-dots with pH-sensitive PL exhibit significant promise for the monitoring of pH changes in living cells, tissues, and living animals, proving they can serve as a useful research tool for biomedical studies.

## Conclusions, Challenges, and Perspective

Since the first discovery in the separation of single-walled carbon nanotubes in 2004 (Xu et al., 2004), C-dots have quickly caused much attention because of the unique PL properties, excellent stability, good biocompatibility, and low biological toxicity. In the past years, the applications of C-dots have rapidly penetrated from materials science to biomedicine, catalysis, energy, environment, and other fields. Although the development of C-dots is so fast, research in various aspects is still in its infancy. The pH sensitivity of C-dots has gradually attracted widespread attention in recent years. In addition, because of the small particle size, good biocompatibility, and photostability, C-dots show great potential in pH sensing, especially in intracellular and *in vivo* pH sensing. In this review, we outline the pH sensitivity property, the pH-sensitive PL mechanism, and the latest application research progress of C-dots. Although C-dots overcome many of the shortcomings of traditional pH probes, there are still some exciting challenges: (1) the PL pH sensitivity and response range of C-dots reported cannot meet the requirement of practical applications. Development of synthetic methods to prepare pH-sensitive C-dots with desired pH response range and sensitivity is a key issue that needs to be solved urgently. (2) The pH-sensitive mechanism of C-dots PL is mostly based on the structures on the C-dots surface, which may have prototropic equilibrium or interact with H^+^/OH^−^. However, the specific groups or structures that should take responsibility to pH sensitivity are still unrevealed. The understanding of the effect of a specific structure or group to the pH sensitivity of C-dot is often controversial. In order to address this issue, it is vital to develop a more specific method to quantitatively regulate specific groups on the surface of C-dots to study the origin of pH-sensitive properties of C-dots, clarify the pH-sensitive fluorescence mechanism, and further propose practical methods for regulating the pH-sensitive fluorescence properties of C-dots. (3) The structures of C-dots synthesized by different raw materials or different methods are very different from each other. When investigating the pH sensitivity PL mechanism of C-dots, it is necessary to consider them differently. The structure contributing to the pH sensitivity may be various for C-dots with different structures because their PL mechanisms are different. Solving these issues will greatly promote the application of the pH-sensitive C-dots.

## Author Contributions

CL wrote the paper. FZ, JH, WG, and MZ revised the paper. All authors contributed to the article and approved the submitted version.

## Conflict of Interest

The authors declare that the research was conducted in the absence of any commercial or financial relationships that could be construed as a potential conflict of interest.
